# Application of contrast-enhanced ultrasound in diagnosis and grading of bladder urothelial carcinoma

**DOI:** 10.1186/s12880-024-01199-3

**Published:** 2024-01-25

**Authors:** Hui-ping Zhang, Rong-xi Liang, Xue-ying Lin, En-sheng Xue, Qin Ye, Yi-fan Zhu

**Affiliations:** Department of Ultrasound, Fujian Medical University Union Hospital, Antai Street & Xin Quan Road 29, Fuzhou, Fujian Province China

**Keywords:** Bladder tumor, Bladder urothelial carcinoma, Contrast-enhanced ultrasound, Grading

## Abstract

**Purpose:**

To explore the application of contrast-enhanced ultrasound (CEUS) for the diagnosis and grading of bladder urothelial carcinoma (BUC).

**Methods:**

The results of a two-dimensional ultrasound, color Doppler ultrasound and CEUS, were analyzed in 173 bladder lesion cases. The ultrasound and surgical pathology results were compared, and their diagnostic efficacy was analyzed.

**Results:**

There were statistically significant differences between BUC and benign lesions in terms of color blood flow distribution intensity and CEUS enhancement intensity (both *P* < 0.05). The area under the time-intensity curve (AUC), rising slope, and peak intensity of BUC were significantly higher than those of benign lesions (all *P* < 0.05). The H/T (height H / basal width T)value of 0.63 was the critical value for distinguishing high- and low-grade BUC, had a diagnostic sensitivity of 80.0% and a specificity of 60.0%.

**Conclusion:**

The combination of CEUS and TIC can help improve the diagnostic accuracy of BUC. There is a statistically significant difference between high- and low-grade BUC in contrast enhancement intensity (*P* < 0.05); The decrease of H/T value indicates the possible increase of the BUC grade.

## Introduction

Epithelial bladder tumors are the most common type of urinary system tumors, its incidence rate accounts for about 3% of all cancers [1]. Bladder urothelial carcinoma (BUC) is the most common malignant epithelial tumor, accounting for approximately 90% of bladder cancers [2–5]. In 2016, the World Health Organization classification of urological and male genital tumors classified bladder and urinary tract epithelial tumors into the following four categories: papilloma, urothelial papilloma with low malignant potential, low-grade BUC, and high-grade BUC [6]. Papillary tumors are benign and papillary urothelial tumors with low malignant potential are borderline tumors, both of which have a relatively good prognosis [7]. Although both low- and high-grade urothelial carcinomas are malignant epithelial tumors, the risk of progression of high-grade BUC is five times higher than that of low-grade BUC, which often requires more active treatment such as cc and postoperative perfusion chemotherapy [3, 8].Therefore, accurate preoperative diagnosis and BUC grading is imperative in the selection of clinical operation methods and the prognostic evaluation.

Currently, accurate grading of BUC is highly dependent on the pathological examination of surgical specimens. Although cystoscopic biopsy can provide preoperative grading information, it is invasive and expensive, and there is a 5 to 15% risk of urinary tract, such as urethral stricture which is not easy for patients to accept [6]. Additionally, cystoscopic biopsy has a misdiagnosis rate of 20–80% [4]. Computed tomography (CT) and magnetic resonance imaging (MRI) play an important role in the preoperative grading and staging diagnosis of BUC; however, CT cannot display the detailed structure of the bladder wall and accurately evaluate the depth of bladder wall infiltration, and MRI may be over-staged [3]. Ultrasonography is simple and easy to perform and is not affected by cystitis, massive hematuria, urethral stricture, or other factors [9]. Some scholars have identified that ultrasound has more advantages in diagnosing BUC without muscular invasion than other imaging modalities [10].

Contrast-enhanced ultrasound can clearly and dynamically observe the blood flow perfusion and distribution of the mass, more accurately identify the benign and malignant of the mass, help find out whether the tumor invades the surrounding tissue, and overcome some limitations of Doppler ultrasound which is poor in detecting low speed and small blood flow [9, 11]. By detecting the harmonic echo signal generated by the microbubbles, inhibiting the strong echo signal of the surrounding tissue, can improve the display rate of microvessels, and at the same time, the conventional intensity ultrasound can destroy the microbubbles [12].The microcirculation blood flow can be measured quantitatively by calculating the refilling process of local tissue microbubbles after destruction [13]. The contrast agent is discharged through the lung after it lasts for about 8 min in the human body, so contrast ultrasound can be used in patients with renal function damage [13, 14]. Some studies have identified that the CEUS is more sensitive than CT in detecting weak blood flow [15]. In recent years, CEUS technology has been widely used in the diagnosis of various diseases including liver and kidney disease [16]. Zhang et al [14] analyzed the CEUS characteristics of renal pelvis transitional cell carcinoma and its relationship with microvessel density, and determined that the peak intensity of CEUS can more effectively evaluate the stage and grade of renal pelvis urothelial carcinoma, and for advanced and high-grade renal pelvis urothelial carcinoma, the peak intensity of CEUS was positively correlated with tissue microvessel density. There are presently only a few studies that report about the role of quantitative analysis of CEUS characteristics in the diagnosis and grading of BUC [6, 17]. In this study we compared and quantitatively analyzed the CEUS characteristics of BUC and benign lesions of the urinary tract as well as the different pathological grades of BUC. In addition, we carried out comparative research on the two-dimensional morphology of different grades of BUC, aiming to help improve the diagnostic accuracy of BUC and provide more information on its grading diagnosis.

## Materials and methods

### Research object

The study participants were patients with bladder disease, diagnosed by surgery and pathology, who were admitted to our hospital from January 2019 to December 2022.

The patient inclusion criteria were as follows: (1) complete clinical data (2) signed informed consent (3) Postoperative pathology obtained after complete resection of the tumor or radical cystectomy.

The exclusion criteria were as follows: (1) patients with severe respiratory diseases; and (2) the final pathological results were urachal carcinoma, metastatic carcinoma, and other bladder malignancies except urothelial carcinoma.

Ultimately, 173 patients with bladder disease were included in this study. A total of 134 patients (77.5%, 134/173) were treated for painless gross hematuria or microscopic hematuria, frequent urination, urinary urgency, painful urination, and other bladder irritations. Two patients were treated for lower abdominal pain and increased nocturia, and 37 patients without any symptoms. The baseline data of 173 patients are shown in the Table 1. All patients were examined using a two-dimensional ultrasound, color Doppler ultrasound, and CEUS before surgery. Written informed consent was obtained from all the patients prior to the contrast ultrasound.

**Table 1:** Basic clinical data and lesion shape distribution of patients with bladder diseases

	High-grade BUC	Low-grade BUC	Benign lesion	P
Number of cases	103	55	15	
Lesion morphology nodular	97	55	14	0.18
Diffuse type	6	0	1	
Age	65.44 ± 11.14	58.60 ± 11.79	54.68 ± 11.84	0.00001
Male/Female	86/17	44/11	11/4	0.60
Single /Multiple	71/32	41/14	14/1	0.13

### Instruments and methods

#### Instrument

A Philips Epiq 7 (Bothell, USA) color Doppler ultrasound diagnostic instrument was used in this study. The probe model was C5-1, the frequency was 3 ~ 5 MHz, the color Doppler frequency was 3.0 ~ 3.5 MHz, the color blood flow range was 3.9 cm/s, and the CEUS used a low mechanical index (MI) of 0.05–0.08. The contrast agent used was SonoVue(Bracco Corp). The preset CEUS conditions were the same for all patients.

#### Methods

Ultrasound examination is performed when the patient’s bladder volume is 200–300 ml, after locating the bladder lesions, we noted the location of the mass, number, size, boundary, echo intensity, and the boundary with the surrounding tissues. In the case of multiple lesions, we focused on the largest lesions. We recorded the basal width T and height H of the lesions, and then calculated the H/T ratio (the basal width of the lesions with diffuse thickening was measured by curve length measurement). Color Doppler flow imaging (CDFI) was used to observe the distribution and intensity of blood flow. The best contrast plane was selected, and in the dual-amplitude contrast display mode we injected 1.2 ml contrast agent microbubble suspension (concentration:5 ml of normal saline injected into 59 mg SonoVue and fully vibrated) through the superficial vein in the patient’s forearm. After the contrast agent was injected, the timer on the instrument panel was immediately started and dynamic storage key was pressed at the same time. During the entire contrast administration process, the patient’s position and the probe position was kept unchanged. To clearly observe the distribution intensity as well as the fading process of the contrast agent entering the bladder wall and the lesion, we guided the patient to take even and slow breaths for 3 min. Then, using the QLAB image processing software, we drew a TIC curve of a 5 mm^2^ representing the region of interest (ROI). During the entire imaging process, we ensured that the lesions ROI remained within the lesion, we recorded the acceleration time (AT), the peak time (PT), the area under the curve (AUC), the rising slope, and the peak intensity. The data was stored in the instrument in DICOM format for analysis and use. None of the patients in this study experienced any discomfort or allergies.

#### Image analysis and judgment standard

CDFI was used to observe the distribution and intensity of blood flow in the lesion. In case of multiple lesions, the largest lesion was observed. The signal intensity of the blood flow in the lesion was divided into “rich blood flow” (≥ 5 point blood flow, two or more longer blood vessels, and the blood flow is reticular or disorderly dendritic), “less blood flow” (< 5 point blood flow, short rod or only one clear long blood vessel and its length can be close to or exceed the radius of the mass), “no obvious blood flow” (no blood flow signal was found in the mass and at the edge) [6]. Contrast-enhanced ultrasound: observed the contrast agent perfusion condition and intensity of the focus, and compared the contrast agent perfusion intensity of the focus with that of the surrounding normal bladder wall. If it was stronger than (or equal to)/lower than the normal bladder wall, it was “high enhancement/low enhancement”, and if there was no contrast agent filling inside the focus, it is “no enhancement”.

### Statistical methods

SPSS19.0 statistical software was used for statistical analysis. The S-W normality test was used for the normality test. The measurement data of normal distribution was expressed in ‾*X ± S*, and the independent sample *t-*test was used between groups; Non-normal distribution measurement data were expressed in *M (Q1, Q3)*. Mann-Whitney *U* test and binary logistic regression analysis were used between groups. The counting data were expressed in frequency and analyzed by Fisher’s test. The difference was statistically significant (*P* < 0.05). The corresponding ROC curve was drawn to evaluate the diagnostic value of H/T value, and the sensitivity and specificity corresponding to the area under the curve and the critical value were calculated.

## Result

### Pathological results

**Fig. 1:**
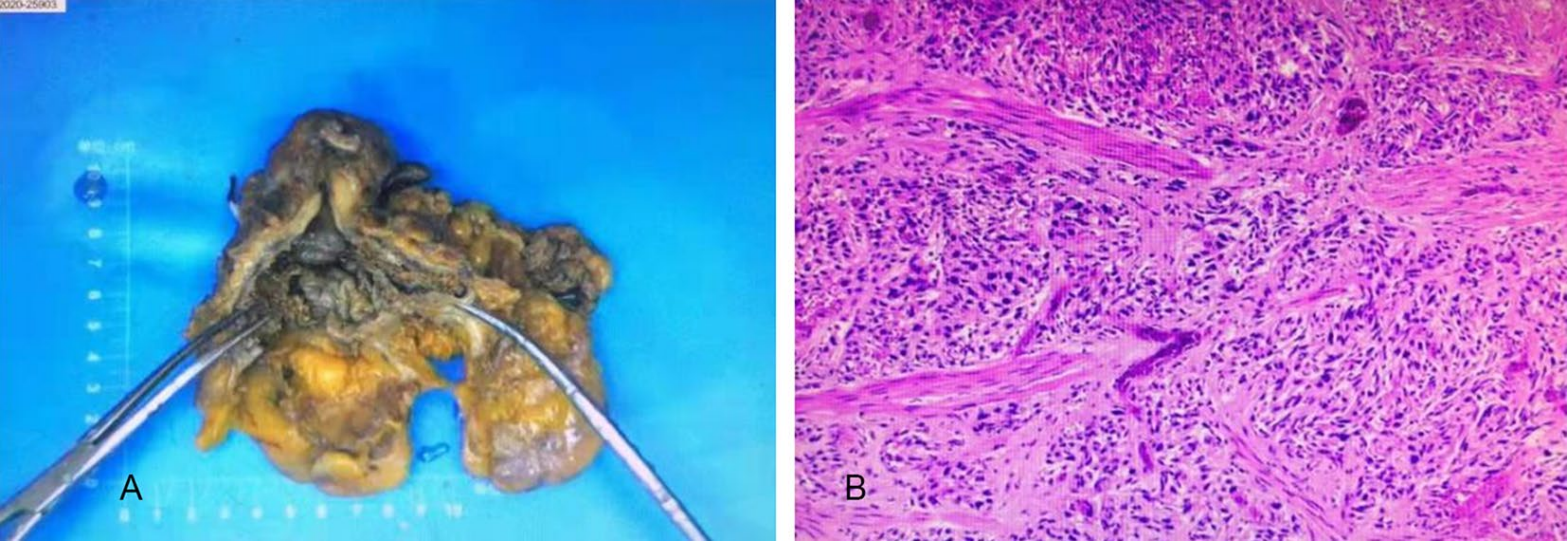
General pathology of high-level BUC. **(A)** High-grade BUC gross specimen. **(B)** Microscopically, tumor cells are diffusely distributed in patches, infiltrating the muscle layer, with hyperchromatic nuclei, inconsistent sizes, inconspicuous nucleoli, and obvious mechanical damage in some regions

**Fig. 2:**
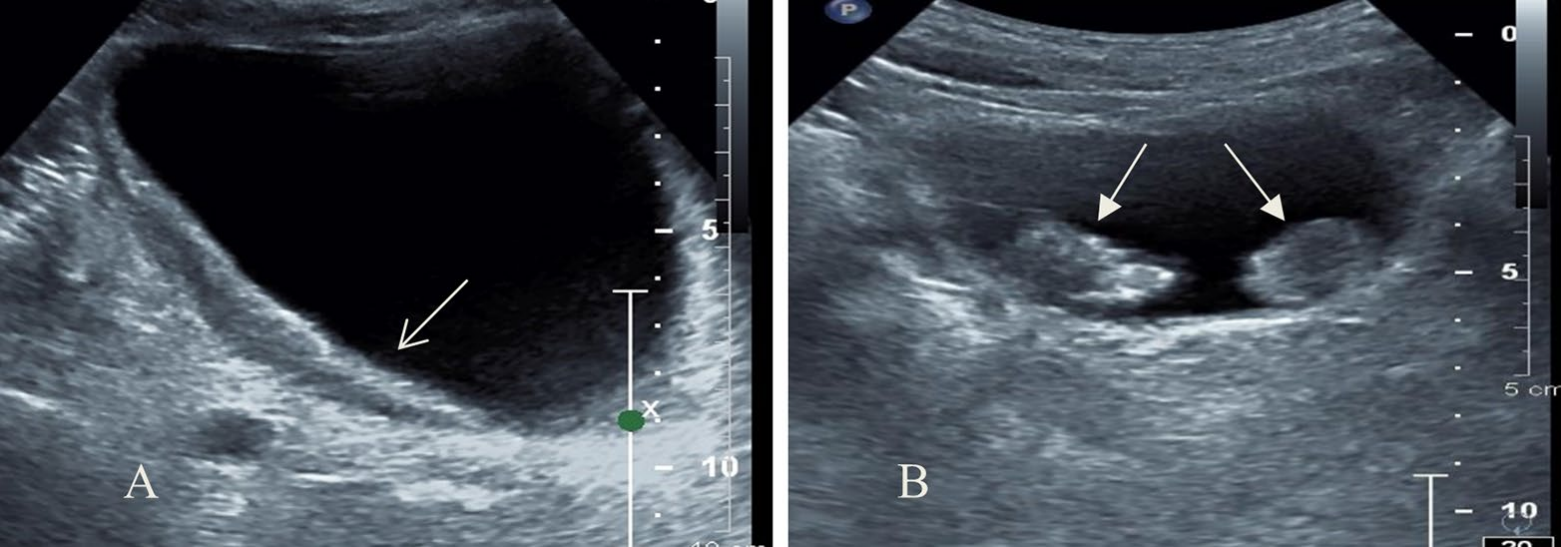
Lesion morphology. **(A)** Diffuse thickening of bladder wall can be seen by two-dimensional ultrasound (arrow). **(B)** Multiple hypoechoic nodules on the bladder wall protrude to the bladder (arrow)

Among 173 patients with bladder lesions (Fig. 1), the largest lesion was about 13.2 cm × 7.9 cm, the smallest was about 0.3 cm × 0.3 cm, the distribution of lesion morphology (Fig. 2) was shown in Table 1.

### Two-dimensional ultrasound, CDFI and CEUS examination

#### Comparison of color doppler blood flow distribution and contrast-enhanced ultrasound intensity

Multiple lesions were the largest lesions observed, and the largest lesions in this study were all lesions with the richest blood flow. Comparison of color Doppler blood flow distribution intensity and CEUS enhancement intensity between the high- and low-grade BUC groups and the benign bladder lesion group (Table 2).

**Table 2:** Comparison of color Doppler blood flow distribution intensity and CEUS enhancement intensity between high- and low-grade BUC group and bladder benign lesion group (number of cases)

Group	Blood Flow Distribution Intensity			CEUS Enhancement Entensity		
	Rich	Less	No	High	Low	NO
High-grade BUC	45	29	29	97	6	0
Low-grade BUC	10	23	22	39	16	0
Benign lesion	0	6	9	9	6	0
P1	0.015			0.000057		
P2	0.006			0.009		

*Note* (P1 was the comparison value between benign and malignant groups, and P2 was the comparison between high- and low-grade BUC group)

#### Diagnostic accuracy of two-dimensional ultrasound and CEUS

**Fig. 3:**
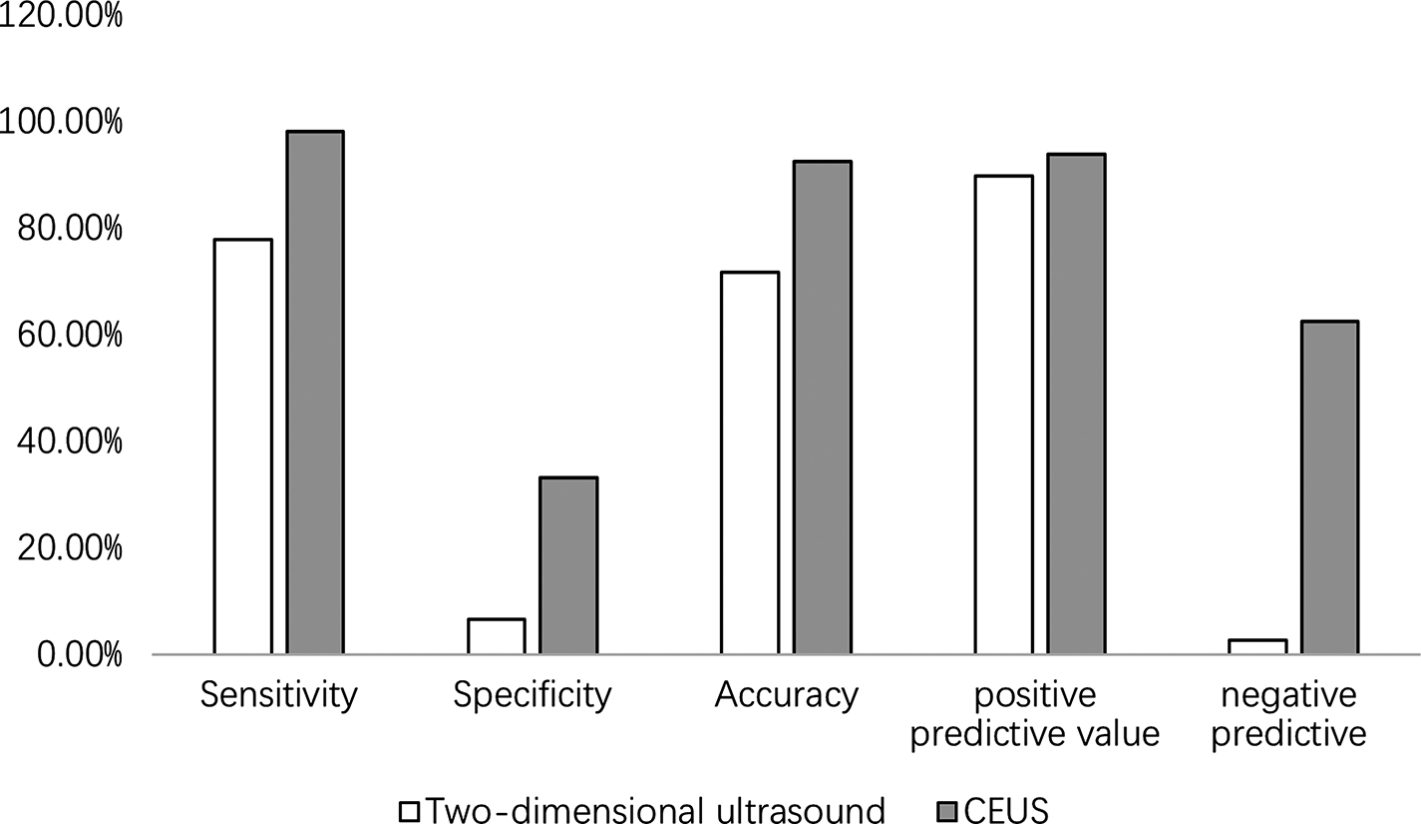
The accuracy, specificity, negative predictive value, and positive predictive value of two-dimensional ultrasound and CEUS

Compared with the results of pathological diagnosis, 124 cases were diagnosed accurately by two-dimensional ultrasound, 49 cases were misdiagnosed (32 cases were not diagnosed qualitatively, 8 benign cases diagnosed as malignant and 9 malignant cases diagnosed as benign), CEUS accurately diagnosed 160 cases, misdiagnosed 13cases (10 benign cases diagnosed as malignant and 3 malignant cases diagnosed as benign), and the accuracy, specificity, negative predictive value, and positive predictive value of two-dimensional ultrasound and CEUS were compared (Fig. 3).

#### TIC curve data analysis results

**Fig. 4:**
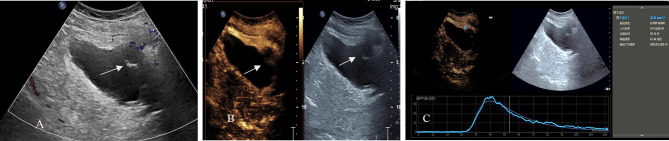
CDFI and CEUS of high-grade BUC. **(A)** High-grade BUC, no obvious blood flow signal in the focus (arrow). **(B)** CEUS examination showed “low enhancement” (arrow). **(C)** TIC curve of high-grade BUC lesions

The TIC curves were drawn for 158 cases of BUC (Fig. 4) and 15 cases of benign lesions. The TIC data (AT, PT, AUC, rising slope, and peak intensity) of benign bladder lesions and BUC were recorded, and the TIC curve data of the two groups were compared (Table 3). Further binary logistic regression analysis of the AUC, rising slope, and peak intensity showed that they were not independent risk factors for BUC (*P >* 0.05) (Table 4), and the diagnostic threshold could not be obtained.

**Table 3:** *M (Q1, Q3)*, *P* value and *t/Z* value of PT, AUC, rising slope and peak intensity

Group	AT(s)	PT(s)	AUC (linear/s)	Ascending slope (Linear/s)	Peak intensity (Linear)
BUC (*n* = 158)	22.5 ± 6.2	32.3 (27.0, 38.8)	3121.3 (1191.9, 7836.2)	12.5 (4.4, 40.0)	94.2 (36.5, 289.6)
Benign (*n* = 15)	23.4 ± 6.4	31.5 (28.0, 34.9)	1182.0 (752.6, 3149.4)	5.5 (2.2, 15.9)	48.6 (16.8, 79.4)
*t/Z*值	0.538	-0.14	-2.38	-2.19	-2.19
*P*	0.591	0.888	0.017	0.029	0.08

**Table 4:** Binary logistic regression analysis of AUC, rising slope and peak intensity

Parameter	Regression Coefficient	Standard Error	Wald χ2	P	OR	95%CI
*AUC*	0.000	0.000	1.596	0.207	1.000	1.000 ~ 1.001
Rising slope	0.004	0.032	0.019	0.891	1.004	0.943 ~ 1.069
Peak intensity	-0.004	0.009	0.178	0.673	0.996	0.980 ~ 1.013

#### Analysis results of the ratio (H/T) of high- and low- grade BUC focus height to base width

**Fig. 5:**
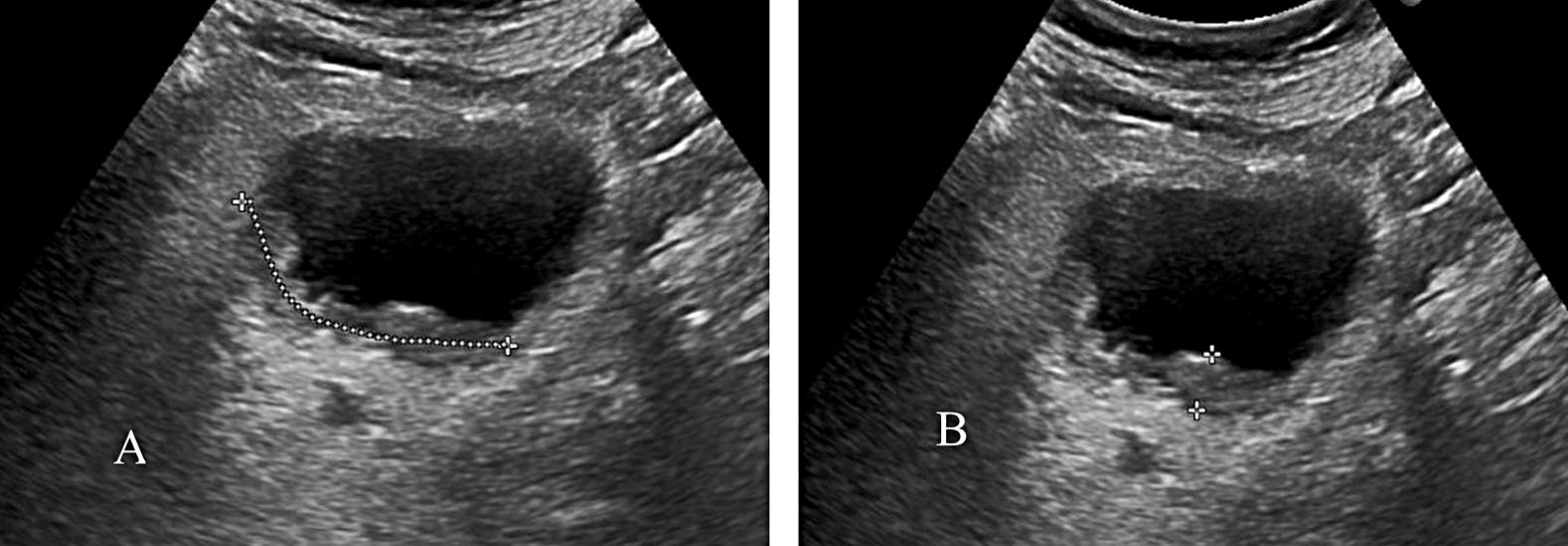
Measurement mode of diffuse lesions. **(A)** Measure the base width (T) of the focus in diffuse thickening cases by curve measurement. **(B)** Measure the height of the focus (H)

**Fig. 6:**
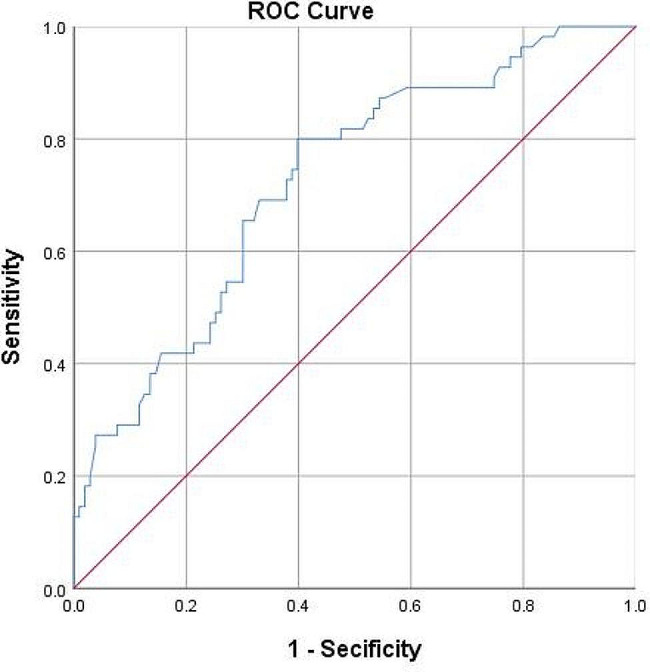
ROC curve of H/T value of high and low grade BUC

Among 158 cases of BUC, 55 cases of low-grade tumors and 103 cases of high-grade tumors were identified using two-dimensional ultrasound. The H/T values of both groups (Fig. 5) were not normally distributed (*P* < 0.05). *M (Q1, Q3) in the* low-grade group was 0.72 (0.64,1.00), and *M (Q1, Q3) in the* high-grade group was 0.57 (0.39, 0.74). There was a statistically significant difference in the H/T values between the two groups (*Z*=-4.638, *P* = 00004, *P* < 0.05). Furthermore, we performed binary logistic regression analysis on the gender, age, and H/T values of high- and low-grade groups and the results showed that the H/T value and age was an independent risk factor for diagnosing high-grade BUC (Table 5). Therefore, the ROC curve of the H/T values can be further drawn (Fig. 6). The AUC was 0.72, optimal critical value was 0.63, sensitivity corresponding to the optimal critical value was 80.0%, and specificity was 60.0%.

**Table 5:** H/T binary logistic regression analysis of high-and low-grade BUC

Parameter	Regression Coefficient	Standard Error	Wald χ^2^	P	OR	95%CI
H/T	-3.346	0.773	18.743	0.000015	0.035	0.008 ~ 0.160
Age	0.066	0.017	14.419	0.000146	1.068	1.033 ~ 1.106
Gender	-0.156	0.505	0.095	0.757	0.856	0.318 ~ 2.300

#### Analysis results of TIC curve data of high- and low-grade BUC

We compared and analyzed the AT, PT, AUC, rising slope, and peak intensity of 103 high-grade BUC cases and 55 low-grade BUC cases (Table 6).

**Table 6:** *M (Q1, Q3)*, *P* and *Z* values of AT, PT, AUC, rising slope and peak intensity of high-and low-grade BUC groups

Group	AT(s)	PT(s)	AUC (linear/s)	Ascending slope (Linear/s)	Peak intensity (linear)
High-grade BUC (*n* = 103)	22.1 ± 5.9	32.0 (27.4, 38.8)	3152.9 (1239.2, 7807.4)	12.4 (4.5, 35.1)	75.4 (40.0, 285.2)
Low-grade BUC (*n* = 55)	22.8 ± 6.4	32.6 (26.0, 39.0)	3089.6 (1004.9, 7922.7)	13.4 (3.9, 50.0)	120.5 (33.3, 372.2)
*t/Z*	-0.649	-0.261	-0.363	-0.089	-0.199
*P*	0.517	0.794	0.716	0.929	0.842

## Discussion

Bladder urothelial carcinoma (BUC), also known as transitional epithelial carcinoma, is the most common malignant tumor of the urinary tract, and accounts for almost 4% of all malignant tumors [18, 19]. Its incidence rate is increasing annually, and it can easily recur. It is mostly observed in middle-aged and elderly men [2]. Tang et al. used the optimal cutoff values for age in their study, and revealed that the survival rates of patients with high grade BUC worsened with increasing age, and it was suggested that age is a strong and independent risk factor for the high graded BUC [20], and our study also suggest that age is an independent risk factor of high grade BUC. Because BUC is categorized into high- and low-grade and different grades of BUC have different biological characteristics [6]. The higher the grade, the higher the degree of malignancy, the faster the tumor progression, the greater the possibility of metastasis, and the worse the prognosis [21]. High- and low-grade BUC have different clinical treatment methods, the major treatment of low-grade BUC is the complete resection of the tumor followed by the use of intravesical adjuvant, and radical cystectomy followed by neoadjuvant chemotherapy is the current standard treatment for high-grade BUC [22]. Therefore, an accurate preoperative diagnosis and histological grading of BUC are imperative. Ultrasound has the advantages of simplicity, ease of operation, and repeatability [9]. Some scholars [10] have demonstrated that ultrasound has more advantages than other imaging examinations in the diagnosis of BUC without muscular invasion and is the preferred imaging examination method for BUC.

The sonogram of bladder BUC mostly showed nodular protruding into the cavity [23]. In this study group, 95.9% showed nodular protrusion into the cavity, only 7 cases showed diffuse thickening, of which 6 (85.7%) were high-grade BUC. Most patients with BUC are treated for painless gross hematuria, the incidence of BUC was 11.0% in macroscopic hematuria cases [24]. Frequent urination, painful urination, dysuria, urinary retention, and lower abdominal masses are late symptoms [25]. In this study, 77.5% of the patients had gross hematuria and bladder irritation symptoms. Therefore, if patients have diffuse thickening of the bladder wall on ultrasound and painless gross hematuria, they should be highly alert to the possibility of high-grade BUC. Ultrasonography is the first choice for screening BUC [24]. Bladder cancer is a vascular-rich tumor with a significantly enhanced arterial phase [26]. In this study, BUC is mostly of less to rich blood flow signals, while benign lesions are mainly of less to no blood flow signals. There are statistically significant differences in blood flow distribution intensity between BUC and benign bladder lesions as well as BUC with different histological grade (*P* < 0.05). It is worth noting that the ultrasound contrast agent is a blood pool tracer, which is not easy to penetrate into the extracellular space through the blood vessel wall [12]. Therefore, the enhancement intensity of the focus is closely related to the tumor vascular density [17]. CEUS can improve the diagnostic sensitivity of tumors rich in blood vessels, especially those with benign and malignant changes that overlap between the blood flow distribution and the focus with little to no blood flow signal, CEUS is helpful for distinguishing between benign and malignant bladder tumors [6, 16].

The vascular lumen of the BUC is narrow, the wall is thin, the endothelium is incomplete, and there are no mature smooth muscle cells or nerve endings [17]. These characteristics hinder the contraction and relaxation of blood vessels, resulting in enhanced intra-arterial perfusion of the tumor [17]. In this study, 86.1% of BUC lesions were highly enhanced, and only 60.0% were highly enhanced in benign lesions, consistent with the pathological changes in blood vessels. There was a statistically significant difference in the CEUS enhancement intensity of different grades of BUC (*P* < 0.05). In this study, BUC was considered positive, and other benign lesions were considered negative for analysis. The results showed that the diagnostic sensitivity, specificity, accuracy, positive predictive value, and negative predictive value of CEUS for BUC were 98.1%, 33.3%, 92.5%, 93.9%, and 62.5%, respectively, which were higher than those of two-dimensional ultrasound. CEUS can accurately reflect the vascular characteristics of BUC, and the combination of CEUS and CDFI can further improve the accuracy of preoperative diagnosis.

Microangiogenesis is an important factor in the growth and metastasis of BUC [6], and Li et al. [17] believe that TIC parameters can objectively and quantitatively analyze the perfusion of contrast media in bladder tumors and reflect their microvascular structure [17]. In this study, the AUC, rising slope, and peak intensity of BUC were higher than those of benign bladder lesions, and the difference was statistically significant (*P* < 0.05), which is related to the existence of a large number of microvessels in BUC that increase the perfusion of contrast media. However, AT and PT were significantly affected by individual heart, lung function, and patient characteristics, and there was no statistically significant difference between the two groups (*P* > 0.05). Therefore, when drawing the TIC curve, the AUC, rising slope, and peak intensity of the lesions were significantly increased, which would help to improve the diagnostic confidence of BUC. However, further binary logistic regression analysis showed that AUC, rising slope, and peak intensity were not independent risk factors for BUC (*P* > 0.05), and the diagnostic threshold could not be obtained. Further studies with larger sample sizes are warranted. Simultaneously, Li et al. [17] studied the role of TIC parameters in the diagnosis of BUC grading. They believe that the peak intensity of high-grade BUC is higher than that of low-grade BUC, the contrast fading time of low-grade BUC is lower than that of high-grade BUC, and the half-slope of high-grade BUC is lower than that of low-grade BUC. The reason is that in high-grade BUC, the blood vessel develops rapidly, the lumen is small, the wall is thin, the endothelium is incomplete, and there are no smooth muscle cells or nerve endings, leading to dysfunction of vasoconstriction and relaxation, which may increase blood perfusion in the arterial phase. The microvessel density of high-grade BUC is higher than that of low-grade BUC, while the vessels of low-grade BUC are relatively regular, making the peak intensity of high-grade BUC angiography stronger than that of low-grade BUC. At the same time, there are more arteriovenous fistulas in the neovasculars in low-grade BUC, and the degree of vascular curvature and interstitial edema is lower. With an increase in the degree of grading, the degree of neovascularization distortion and interstitial edema increases, which slows down the scouring of the intravascular contrast agent, resulting in the contrast fading time of high-grade BUC being longer than that of low-grade BUC, and the half-slope being lower than that of low-grade BUC [6, 17]. However, in this study, there was no statistically significant difference between the TIC parameters of BUCs with different histological grades (*P* > 0.05), which may be related to the small sample size. Due to software limitations, the study did not record and analyze the half-time and half-slope of the lesions, and further improvement of the sample size and software is needed.

Ozden et al. [3] performed a comparative analysis of the tumor height, the contact length between the tumor and bladder wall, and the ratio of invasive bladder cancer to non-invasive bladder cancer, and concluded that the tumor height, the contact length between the tumor and bladder wall, and the ratio of the two are closely related to the depth of bladder cancer invasion. The contact length was more than 41.5 mm, and the ratio of the two was less than 0.605, which is the critical value for distinguishing superficial and invasive bladder cancer. Miyamoto et al. [27] also studied the relationship between the maximum contact length of the tumor and the recurrence of prostate cancer and reported that the maximum contact length of the tumor was an independent risk factor for predicting the biochemical recurrence of prostate cancer. In addition, the recurrence and metastasis of BUC are closely related to its grade. The higher the grade, the higher the degree of malignancy, the faster the tumor progression, the greater the possibility of metastasis, and the worse the prognosis [21]. To study the relationship between the maximum contact length of BUC and the bladder wall and its recurrence and metastasis, the H/T values of high- and low-grade urothelial carcinoma were compared and analyzed. The results showed that the difference between the two groups was statistically significant (*P*; that is, the smaller the H/T value, the higher the BUC grade). The larger the H/T value, the lower the BUC grade. Therefore, the H/T ratio can provide relatively reliable diagnostic information for the pathological grade of BUC.

This study has several limitations: As mentioned previously, flat lesions, small lesions and multiple lesions may not be seen easily and the 3D ultrasound may help in some cases. The sample size of this study was limited, and no partial diagnosis of BUC was performed. The role of CEUS in BUC staging still needs to be further explored; because of the software limitations, the half-time and half-slope of the lesions were not recorded and analyzed, which requires further improvement.

## Conclusion

The combination of CEUS and TIC curve can help improve the diagnostic accuracy of BUC. There is a statistically significant difference between high- and low-grade BUC in contrast enhancement intensity (*P* < 0.05), but there is no statistically significant difference between the parameters of TIC curve (*P* > 0.05); The decrease of H/T value indicates that the possibility of increasing BUC grade is increased.

## Data Availability

The data that support the findings of this study are available on request from the corresponding author. The data are not publicly available due to privacy or ethical restrictions.
